# Secondary Publication: Proposal for Points of Consideration for Pluripotent Stem Cell Culture

**DOI:** 10.1007/s11626-024-00863-w

**Published:** 2024-03-12

**Authors:** Takashi Aoi, Isao Asaka, Hidenori Akutsu, Yuzuru Ito, Ken Kataoka, Yasunari Kanda, Hajime Kojima, Yuko Sekino, Hirofumi Suemori, Masato Nakagawa, Kazuaki Nakamura, Yukio Nakamura, Makiko Fujii, Miho Furue, Daiju Yamazaki, Japanese Working Group for Consideration of Good Cell Culture Practice

**Affiliations:** 1 Division of Stem Cell Medicine, Graduate School of Medicine, Kobe University, Kobe, Hyogo 650-0017 Japan; 2 Department of Fundamental Cell Technology, Center for iPS Cell Research and Application, Kyoto University, Kyoto, 606-8507 Japan; 3 Center for Regenerative Medicine, National Center for Child Health and Development, Tokyo, 157-8538 Japan; 4 Faculty of Life and Environmental Sciences (Bioindustrial Sciences), University of Tsukuba, Tsukuba, Ibaraki 305-8972 Japan; 5 Department of Life Science, Faculty of Science, Okayama University of Science, Okayama, 700‑0005 Japan; 6 Division of Pharmacology, National Institute of Health Sciences, Kawasaki, Kanagawa 210-0821 Japan; 7 Japanese Center for the Validation of Alternative Methods, National Institute of Health Sciences, Kawasaki, Kanagawa 210-0821 Japan; 8 Graduate School of Agricultural and Life Sciences, The University of Tokyo, Bunkyo-Ku, Tokyo, 113-8657 Japan; 9 Division of Clinical Basis for ES Cell Research, Institute for Frontier Life and Medical Sciences, Center for Human ES Cell Research, Kyoto University, 53 Kawahara-Cho, Shogoin, Sakyo-Ku, Kyoto, 606-8507 Japan; 10 Department of Life Science Frontiers, Center for iPS Cell Research and Application, Kyoto University, Kyoto, 606-8507 Japan; 11 Department of Pharmacology, National Research Institute for Child Health and Development, Tokyo, 157-8535 Japan; 12 Cell Engineering Division, RIKEN BioResource Research Center, Tsukuba, 305-0074 Japan; 13 Department of Genomic Oncology and Oral Medicine, Graduate School of Biomedical and Health Science, Hiroshima University, Hiroshima, 834-8553 Japan; 14 Laboratory of Stem Cell Cultures, National Institute of Biomedical Innovation, Health and Nutrition, Ibaraki, Osaka 567-0085 Japan; 15 Present Address: Cel-MiM, Ltd., Tokyo, Japan

**Keywords:** Cell culture, Good Cell Culture Practice, Pluripotent stem cell, Diversity

## Abstract

Human pluripotent stem cells, such as human embryonic stem cells and human induced pluripotent stem cells, are used in basic research and various applied fields, including drug discovery and regenerative medicine. Stem cell technologies have developed rapidly in recent years, and the supply of culture materials has improved. This has facilitated the culture of human pluripotent stem cells and has enabled an increasing number of researchers and bioengineers to access this technology. At the same time, it is a challenge to share the basic concepts and techniques of this technology among researchers and technicians to ensure the reproducibility of research results. Human pluripotent stem cells differ from conventional somatic cells in many aspects, and many points need to be considered in their handling, even for those experienced in cell culture. Therefore, we have prepared this proposal, “Points of Consideration for Pluripotent Stem Cell Culture,” to promote the effective use of human pluripotent stem cells. This proposal includes seven items to be considered and practices to be confirmed before using human pluripotent stem cells. These are laws/guidelines and consent/material transfer agreements, diversity of pluripotent stem cells, culture materials, thawing procedure, media exchange and cell passaging, freezing procedure, and culture management. We aim for the concept of these points of consideration to be shared by researchers and technicians involved in the cell culture of pluripotent stem cells. In this way, we hope the reliability of research using pluripotent stem cells can be improved, and cell culture technology will advance.

## Preface

In recent years, cell culture–related technologies have rapidly developed, and cell culture now contributes to a wide range of fields, having expanded beyond basic research to applied areas such as drug discovery and regenerative medicine. Human pluripotent stem cells (hereafter, pluripotent stem cells), such as human embryonic stem (ES) cells (Thomson *et al*. 1998) and human induced pluripotent stem (iPS) cells (Takahashi *et al*. 2007), are expected to be widely used in these areas in the coming decades. Accordingly, the supply of base materials, culture media, and cells has improved, making the culture of human pluripotent stem cells feasible for many laboratories. Culturing pluripotent stem cells is, however, technically demanding, and maintaining their undifferentiated nature requires skill. Also, changes in differentiation potential and instability of differentiation efficiency make it difficult to ensure reproducibility. The first step in obtaining consistent results is to share among researchers and technicians the techniques necessary for maintaining undifferentiated characteristics and the background knowledge and concepts that underpin these techniques. The culture of pluripotent stem cells differs from that of conventionally used somatic cells in many aspects, and even researchers who are experienced and proficient in cell culture need to be aware of the limitations of these cells and their specific culture management/operations. Therefore, to promote the effective use of human pluripotent stem cells, we have compiled a list of items to consider for researchers and technicians who culture these cells.

Note that pluripotent stem cell research is continually developing; therefore, we recommend keeping up to date with the latest information on these points of consideration.

## Points of Consideration for Pluripotent Stem Cell Culture

### Introduction

Pluripotent stem cells are predicted to be used extensively in basic research, regenerative medicine, and drug discovery. However, because the undifferentiated and pluripotent nature of pluripotent stem cells is difficult to control, researchers and technicians should share the basic concepts and fundamental techniques needed for their culture. Different applications call for different perspectives on quality control, but many of the underlying technologies and controls are common to all applications. This proposal summarizes and proposes points to be considered that are common to all experiments and research involving the culture of pluripotent stem cells. The authors previously published “Fundamental Principles of Cell Culture” (Kanda *et al*. 2017) and “Fundamental Principles for Microscopic Observation of Cultured Cells” (Nakamura *et al*. 2017) in Japanese in TCRC by JTCA. The fundamental principles presented in these studies should also be followed in handling pluripotent stem cells. This proposal describes the points to be considered for the appropriate maintenance and culture of undifferentiated pluripotent stem cells for researchers and technicians. For information on the establishment or induction of differentiation of pluripotent stem cells, please refer to other latest information or publications.

### Purpose

To improve the reproducibility and reliability of basic research or research aimed at clinical application using pluripotent stem cells, we list the following fundamental points to be considered for the maintenance and culture of pluripotent stem cells. These points are common considerations that are strongly recommended to be followed by all researchers and workers in pluripotent stem cell culture. Preclinical trials for the purpose of clinical research, as well as basic research, should be conducted appropriately and, in addition to complying with relevant laws and guidelines, the points of consideration described in this proposal follow these conditions. Furthermore, instructors must adhere to this fundamental principle when providing guidance on pluripotent stem cell culture.

### Points of consideration

#### Laws/guidelines and agreements/material transfer agreements (MTAs) must be confirmed before use of pluripotent stem cells is commenced.

When using pluripotent stem cells, such as ES cells and iPS cells, their intellectual property rights, conditions of use, and related laws/guidelines must be confirmed, and each cell must be used within the scope of use defined in these documents (International Stem Cell Banking Initiative 2009; Isasi and Knoppers 2009; Takada *et al*. 2011).

1. Must be used within the scope specified by laws /guidelines.
  For example, pluripotent stem cells have the potential to produce human embryos through germ cell differentiation; however, in Japan, transfer of these human embryos to the uterus is prohibited, and germ cell differentiation and embryo production are strictly regulated by laws and guidelines.^1^ Before conducting cell culture, a research plan must be prepared based on laws/guidelines, and the research must be carried out following the research plan. However, laws/guidelines may be revised; therefore, it is important to organize a system whereby the latest information can be obtained at all times.
2. Must be used within the scope specified by MTAs and other agreements. 

In many cases, pluripotent stem cells are subject to intellectual property rights, and an MTA or other agreement needs to be signed before cells can be obtained. The MTA or other agreement must be checked when using the received pluripotent stem cell lines. The cells must be used within the scope of the written consent given by the provider or the donor.^2^

#### Diversity of pluripotent stem cells

Cell line characteristics are diverse; self-renewal (maintenance in an undifferentiated state) and differentiation capacities vary among cell lines (International Stem Cell Initiative *et al*. 2007; Andrews *et al*. 2005; International Stem Cell Initiative *et al*. 2011). Therefore, it is necessary to understand the characteristics of each cell line before use. The characteristics of pluripotent stem cells may differ among clones; even if the clones are derived from the same donor, attention should be paid to clone divergence.

1. Confirm growth characteristics and differentiation potential from information obtained at cell acquisition.
  If ES cells or iPS cells are obtained from public cell banks, the culture conditions, such as the culture media to be used, should be confirmed by checking the attached cell information sheet,^3^ and the growth characteristics and differentiation potential should also be confirmed. If the cells are obtained from the research community,^4^ information about the cells should be obtained from the provider.
2. Confirm growth and differentiation capacity in the handling institution.
  Differences in culture procedure or reagent lot may alter cell characteristics, such as growth and differentiation potential.^5^ Growth and differentiation potential in the handling institution must be confirmed, referring to the cell information sheet obtained at the time of cell acquisition.
3. Establish assessment criteria for each cell line to be handled.
  Cell characteristics, such as growth rate and differentiation tendency, vary among cell lines (Kajiwara *et al*. 2012; Osafune *et al*. 2008; Yanagihara *et al*. 2016). Assessment criteria for quality control should be established in the handling institution for each cell line to ensure consistent quality at all times^6^ (Kusuda-Furue 2008; Kusuda-Furue 2009; Hirata *et al*. 2011).

#### Culture materials

Materials, such as culture vessels and media that directly contact pluripotent stem cells, greatly influence cell characteristics. Therefore, it is necessary to select and use materials specified in the protocol, taking into consideration differences among manufacturers and lots. It should be noted that cell characteristics, such as proliferation rate and differentiation efficiency of a cell line, can be significantly altered by differences in culture media and culture conditions.

1. Ensure in advance whether the culture vessels to be used continuously suit the culture.
  Culture vessels vary not only in shape and size but also in the composition of the resin, culture surface treatment, air permeability, and other factors, depending on the manufacturer. These differences can alter the adhesion and growth of pluripotent stem cells.
2. Be careful when handling culture media, reagents, and coating materials (e.g., extracellular matrix). 
  Media are the most important material in pluripotent stem cell culture.^7^ The performance of culture media is affected by differences/expiration dates of culture media/additives and changes in pH and other parameters over time after preparation or after opening a container; media must be handled and used with sufficient attention to these points.^8^ The specified storage method in the protocol must be followed because performance changes depending on the storage method and period. In addition, coating of culture surfaces requires attention to the type of coating material used and its concentration, treatment temperature, treatment time, and expiration after coating. Variations in these conditions may alter cell adhesion and affect the characteristics of pluripotent stem cells.^9^
3. Do not inadvertently change feeder cell type.
  Do not inadvertently change feeder cell type when they are used because the composition of factors affecting growth and maintenance of undifferentiated pluripotent stem cells can be changed depending on the origin and preparation/inactivation method of the feeder cells. Attention must also be paid to seeding density in the culture vessel and the time elapsed after seeding, as these factors may also affect the characteristics of the pluripotent stem cells.

#### Thawing procedure

The appropriate thawing procedure varies depending on the composition of the cryopreservation solution; therefore, the specified thawing procedure in the protocol must be followed. Attention must also be paid to the time taken to perform the procedure. After thawing, care must be taken regarding the temperature and time the reagent is in contact with the cryopreservation reagent, because these factors affect cell viability. The number of cells seeded should not be smaller than the specified density in the protocol because the post-thaw survival rate of pluripotent stem cells is often lower than that of common cultured cell lines, and the cell adhesion efficiency and colony formation rate are often even lower than the survival rate.

#### Medium replacement and passaging

Appropriate management of the culture environment is necessary for pluripotent stem cells to properly maintain their undifferentiated state. Users must understand that even a small change in conditions can easily transform pluripotent stem cells into some kind of differentiated cell.

1. Be careful with the timing of media replacement.
  Pluripotent stem cells are susceptible to degradation of growth factors in the culture medium and consumption of nutrients and associated waste accumulation.^10^ The rate of progression of these changes depends on the number of cells; therefore, it is necessary to adjust the frequency and volume of medium replacement according to the increase in the number of cells. In addition, autocrine and paracrine factors may affect cell growth and maintenance of the undifferentiated state. Their effects vary depending on the number of cells; therefore, it is necessary to consider their effects in the early and late stages of culture separately.
2. Be careful with passage timing. 
  The passage schedule should be created, taking into account the timing of medium exchange and the growth of cells. Pluripotent stem cells grow as colonies; as colony size increases, differences appear among colonies or between the center and periphery of a colony (Amit and Itskovitz-Eldor 2012). Passaging should be performed as possible, while the colonies are comparable in size to maintain the homogeneity of the cells.
3. Be careful with the cell dispersion treatment and pipetting.
  Cell lines may differ in their tolerance to cell-dispersing agents and mechanical stress, and passage operation may alter the nature of the cell population. Therefore, it is recommended to avoid prolonged dispersion and excessive pipetting as much as possible (Asaka and Yamanaka 2012).

#### Freezing procedure

It is necessary to freeze the cells carefully so that the original characteristics of the pluripotent stem cells can be maintained.

1. Prepare frozen stock as early as possible after the cells are obtained.
  The freezing tolerance of pluripotent stem cells is often low. As repeated freeze-thawing may result in the selection of cell populations with relatively high freezing tolerance, it is desirable to prepare as many frozen stocks as possible immediately after cell acquisition.
2. Perform freezing appropriately.
  The appropriate thawing method varies depending on the composition of the cryopreservation solution; therefore, the specified thawing procedure in the protocol must be followed, and attention must be paid to the time taken to perform the procedure. The temperature of the reagent and the time it is in contact with the cryopreservation reagent affect cell viability; therefore, it is necessary to control this to ensure a certain level of survival. In addition, the freezing tolerance of pluripotent stem cells varies among cell lines. A preliminary test should be performed before freezing newly established cell lines (Asaka and Yamanaka 2012).

#### Culture management

Pluripotent stem cells need to be handled with the understanding that these cells are prone to change their phenotypes.

1. Keep track of the number of cell divisions in the management plan.
  Although the capacity of pluripotent stem cells to divide is almost infinite, repeated cell division causes changes in characteristics due to cell selection and adaptation, and also increases the risk of mutation occurrence. It is therefore desirable to avoid long-term passaging/maintenance and population doubling level (PDL) should be tracked in the management plan, if possible. If tracking the PDL is difficult, record the number of passages, days of incubation, and split ratio.
2. Test the characteristics of the stem cells on a periodic basis.
  If the cells are to be used continuously for a long period, for example, 10 passages or more after acquisition, characteristics such as cell morphology, growth capacity, expression profile of undifferentiated/differentiated markers, and abnormal karyotype should be periodically checked to confirm that they are maintained. No absolute marker to confirm pluripotency has been discovered; therefore, it is necessary to test multiple markers.
  Note that confirmation by a cell identification test and a mycoplasma test is required if more than 6 mo have passed since acquisition.^11^
  In principle, the culture protocol should not be changed; however, if the culture medium or passaging method is changed for experimental reasons, the above tests should be performed to confirm that the characteristics of the stem cell have not changed.
3. If cell proliferation or morphology is observed to have changed, do not continue to use the cells.
  If the following situations appear during the continuous culture and maintenance of pluripotent stem cells, culture/passage conditions may be inappropriate, or some kind of abnormality may have occurred: many colonies of differentiated cells are observed in the early phase after passaging, many differentiated cells are observed in a colony, or the growth rate is rapidly elevated. In these cases, the cells should be discarded and instead frozen stock prepared early after cell acquisition should be thawed and used as the cell characteristics are considered to have changed. However, if there is an absolute need, collect undifferentiated colonies only and continue the culture.

## Conclusions

We propose the above seven items as “Points of Consideration for Pluripotent Stem Cell Culture.” In culturing pluripotent stem cells, it is always important to accurately judge the state of the cells, taking into consideration that cell characteristics can easily change according to the culture process. Loss of differentiation potential and appearance of differentiated cells greatly affect results using pluripotent stem cells. To appropriately maintain the cells’ undifferentiated nature and pluripotency, it is necessary to carry out culture operations with great care by following the above points.

Previously, the Japanese Tissue Culture Association (JTCA) and the Japanese Society for Alternatives to Animal Experiments (JSAAE), together with outside experts, established a working group and developed “Fundamental Principles of Cell Culture” (Kanda *et al*. 2017) and “Fundamental Principles for Microscopic Observation of Cultured Cells” (Nakamura *et al*. 2017), and these principles were proposed to these JTCA and JSAAE society members. These principles were also disseminated to related academic societies and organizations, and were posted on their websites, as well as on the website of the Japan Agency for Medical Research and Development (AMED). In the proposal, we presented “Points of Consideration for Pluripotent Stem Cell Culture” (Aoi *et al*. 2019) to the members of the Japanese Tissue Culture Association to promote the exchange of information and opinions with the relevant departments of AMED, the Ministry of Health, Labour and Welfare, the Ministry of Economy, Trade and Industry, and the Ministry of Education, Culture, Sports, Science and Technology. Through common recognition and information exchange with other academic societies that have researchers involved in experiments using cell culture, we hope that many researchers and technicians involved in cell culture could understand the aim of these principles and proposal of points of consideration, and that the techniques will be advanced to further develop research using cell culture.

## Footnotes

1 Life Science Portal site of the Ministry of Education, Culture, Sports, Science and Technology provides information on relevant laws/guidelines to be followed when conducting research using human ES cells and human iPS cells. Before research is commenced, it is necessary to be familiar with the contents and to go through the required procedures. http://www.lifescience.mext.go.jp/bioethics/hito_es.html

2 Some cells have limitations in their use, e.g., genome analysis; this point must always be checked.

3 When thawing and culturing cells, proceed under the conditions described in the information sheet. It is not uncommon for products with the same product name to have different characteristics depending on the manufacturer; the manufacturer, catalog numbers, and other information must be checked in detail. Even if it is necessary to change the culture conditions for experimental reasons, first expand the culture under the specified conditions in the protocol and preserve the cells before changing the conditions. In some cases, growth characteristics and differentiation potential are not described in the information sheet. In such cases, the experimenters themselves need to confirm the growth characteristics and differentiation potential at the start of use.

4 Human ES cells can be used only by organizations that have notified the Government of their utilization plans (utilizing institute) in accordance with the “Guidelines on the Distribution and Utilization of Human Embryonic Stem Cells.” In principle, utilizing institutes cannot distribute human ES cells to other organizations. However, this shall not apply to the transfer of differentiated cells derived from human ES cells or the distribution of human ES cells to utilizing clinical institutes. See Articles 7, 23 and 35 of the Guidelines.

5 The growth and differentiation potential of pluripotent stem cells are altered by a variety of influences. Care must always be taken because irreversible changes may occur. Therefore, the information at the time of cell acquisition and the growth status in the laboratory should be tracked.

6 The characteristics of the cell lines should be confirmed before using for research in the laboratory.

7 Serum or serum substitutes, along with growth factors and amino acids, are added to the basic media; otherwise, complete serum-free media prepared by the manufacturer may be used.

8 The concentration described in the paper is a reference value. The appropriate concentration often varies depending on the cell used and the manufacturer/lot of the reagent; therefore, it is necessary to confirm the appropriate concentration under the working conditions. Note that even reagents of the same name have different titers between natural extracts and recombinant forms. In particular, enzymes extracted from natural products should be tested before use because their origins and purification methods vary depending on the manufacturer, resulting in differences in their titers, and also because lot differences can affect the performance.

9 Note that even cell adhesion factors of the same name may differ in their origins and expression systems (in the case of recombinant products); even the same product may have variability because of lot differences.

10 Warming a whole bottle of culture medium leads to deterioration of the medium. It is desirable to calculate the amount of medium needed and warm it up in a tube or other containers.

11 An increasing number of journals require cell line authentication and a negative mycoplasma test result for the cultured cells used at the time of paper submission. Cell identification tests and mycoplasma tests are required if more than 6 mo have passed since cell acquisition (not required within 6 mo of cell acquisition).
